# Supplementary material for: Risk of New-Onset Dementia in Patients with Chronic Kidney Disease on Statin Users: A Population-Based Cohort Study
Source: Biomedicines. 2023 Apr 2;11(4):1073. doi: 10.3390/biomedicines11041073 (PMC10135687; doi:10.3390/biomedicines11041073)
Supplement: Supplementary file 1 [file biomedicines-11-01073-s001.zip › biomedicines-2253286-supplementary.pdf]

Supplemental Table S1

Comorbidity	ICD-9 CM	ICD-10 CM
Diabetes mellitus	250	E08
Coronary heart disease	410-415	I21, 25
Hypertension	401-405	I10-I13, I15
Coronary heart disease	410-415	I21, 25
Dyslipidemia	272	E78
Chronic obstructive pulmonary disease	490, 491, 492, 494, 495, 496	J44
Ischemic stroke	434	I63, I66
Peptic ulcer	533	K27
Dementia	294	F02, F03, F04
Chronic kidney disease	585	N18
Heart failure	428, 402.x1, 403.x1	I50, I10.1, I13.0, I13.2
Peripheral artery disease	443.9	I73.9
